# Early home-based recognition of anaemia via general danger signs, in young children, in a malaria endemic community in north-east Tanzania

**DOI:** 10.1186/1475-2875-5-111

**Published:** 2006-11-20

**Authors:** Frank M Ringsted, Ib C Bygbjerg, Helle Samuelsen

**Affiliations:** 1 Department of International Health, Institute of Public Health, University of Copenhagen, 5, Øster Farimagsgade, DK-1410 Copenhagen K, Denmark

## Abstract

**Background:**

Ethnographic studies from East Africa suggest that cerebral malaria and anaemia are not classified in local knowledge as malaria complications, but as illnesses in their own right. Cerebral malaria '*degedege' *has been most researched, in spite of anaemia being a much more frequent complication in infants, and not much is known on how this is interpreted by caretakers. Anaemia is difficult to recognize clinically, even by health workers.

**Methods:**

Ethnographic longitudinal cohort field study for 14 months, with monthly home-visits in families of 63 newborn babies, identified by community census, followed throughout April – November 2003 and during follow-up in April-May 2004. Interviews with care-takers (mostly mothers) and observational studies of infants and social environment were combined with three haemoglobin (Hb) screenings, supplemented with reports from mothers after health facility use.

**Results:**

General danger signs, reported by mothers, e.g. infant unable to breast-feed or sit, too weak to be carried on back – besides of more alarming signs such as sleeping all time, loosing consciousness or convulsing – were well associated with actual or evolving moderate to severe anaemia (Hb ≤ 5–8 g/dl). By integrating the local descriptions of danger symptoms and signs, and comparing with actual or evolving low Hb, an algorithm to detect anaemia was developed, with significant sensitivity and specificity. For most danger signs, mothers twice as often took young children to traditional healers for herbal treatment, rather than having their children admitted to hospital. As expected, pallor was more rarely recognized by mothers, or primary reason for treatment seeking.

**Conclusion:**

Mothers do recognize and respond to symptoms and danger signs related to development of anaemia, the most frequent complication of malaria in young children in malaria endemic areas. Mothers' observations and actions should be reconsidered and integrated in management of childhood illness programmes.

## Background

In areas with intense malaria transmission, such as the lowlands of north-eastern (NE) Tanzania, anaemia is the most common manifestation of severe malaria [1], peaking at the age of one year, and is a significant risk factor for child hospital mortality [2].

The World Health Organization (WHO) strategy for anaemia detection, under the Integrated Management of Childhood Illness (IMCI) [3], includes algorithms for pallor assessment. However, pallor is not very sensitive for detecting anaemia, even for health professionals [4], wherefore early recognition by additional symptoms and signs by caretakers is desirable.

While fever is recognized as the common manifestation of, even equalled to, malaria, and usually instigates prompt health seeking by caretakers, anaemia is a 'silent burden' [5] hiding behind and aggravating morbidity and mortality. Anaemia may progress even after anti-malarial treatment, and recovery to normal haemoglobin (Hb) levels may take up to six weeks [6]. A study in Kenya [7] of parental recognition of symptoms and signs related to anaemia, reported about 60 % sensitivity and specificity of 'weak body' plus pallor after instruction of caretakers, for detecting moderate-severe anaemia (Hb < 8 g/dl) in infants. Numerous studies on clinical and pathological aspects of malaria in infancy have been undertaken in East Africa, and some ethnographic studies on the local perceptions of malaria and its complication [8-10], but mostly on cerebral manifestations e.g. '*degedege*' [11-14], in spite of anaemia being the most frequent complication [2]. Ethnographic studies suggest that cerebral malaria and anaemia are generally not classified in local knowledge as malaria complications, but as illnesses in their own right [15-17].

To investigate if pallor, weak body and possibly other ''danger signs" were known to caretakers of new-born babies, and which actions were taken against them, and at what stage, a longitudinal study was undertaken in NE Tanzania, with an ethnographic approach.

## Methods and settings

An ethnographic approach was used, aiming at identifying practical knowledge of mothers, i.e. their 'clinical' skills for symptom or sign awareness, and the application of that knowledge at the time of illness [18]. The study was designed to establish confidence of a limited number of informants, followed regularly over 14 months, to allow for sensitive information, such as illness cases attributed to witchcraft and use of healers.

Following a community census in early 2003, covering 2,500 households in Muheza town, Tanga Region, NE Tanzania, in which infants born within the five preceding months of the onset of the high malaria transmission season (in May) were eligible. 111 live-born infants were identified, and followed for a full annual cycle of malaria risk, acknowledging that vulnerability to malaria starts from about the age of four months, when maternal protective antibodies decline. Thus, infants' may include small children up to the age of 18 months. Because of other ongoing research projects involving infants, and migration of mothers to the countryside after delivery or marriage, only 63 infants were eligible to, and accepted inclusion by caretakers, while two had died, reportedly from pneumonia.

For each infant, caretakers, mostly mothers, were interviewed in-depth, in the local joint language, *Kiswahili*, by the principal investigator and a local, respected female interpreter, during one-monthly home-visits of one hour duration, combined with observations of infant, care-takers and social environment. In case of illness, in-between visits took place.

On three occasions, by the end of the long rains, (high transmission period), the short rains, and at the onset of the next long rains, in August, November, and April, respectively, levels of Hb were estimated, by finger-prick blood and HaemoCue^® ^(AB, Sweden), supplemented by passive detection of Hb at health facility in case of clinic visits, as reported by mothers.

Infection and nutritional factors intertwine in anaemia development, but were deliberately not examined in this study, in order to avoid the caretakers being "induced" or the infants "over-researched".

### Ethics

Ethical clearance was granted by the Scientific Committee of the Tanzania National Institute of Medical Research, NIMR, and the Tanzanian Commission for Science and Technology, COSTECH. Mothers volunteered after written consent, and were ensured of the confidentiality of the information they offered, and guaranteed anonymity. The principal investigator (PI) being a trained anthropologist, not a medical doctor, implied that medical investigations and interventions were only undertaken at health facilities and after referral, in response to care-takers' preferences. However, the project offered free access and admission to the local Maternal and Child Clinic (MCH) at/to the local district hospital. Following the procedure of the latter, infants with Hb ≥ 7 g/dl were told to mothers to have 'enough blood', while children with Hb < 7 g/dl, or with difficulties in breathing or prostration during interview were offered referral to hospital.

## Results

### Mothers' concepts and vocabulary related to malaria and anaemia

Mothers' general knowledge of illness symptoms and signs in their babies reflected experience from having another child, previous clinic attendance, school attendance and traditional beliefs. Table 1 lists the *Kiswahili *terms most frequently used by mothers.

**Table 1:** List of most common *Kiswahili *terms used by mothers of Muheza town for malaria related symptoms and signs in their infants.

*Kiswahili *term	English translation
*Homa*	Febrile illness
*Homa kali*	Strong febrile illness
*Homa mara kwa mara*	Febrile illness on-and-off
*Joto*	Heat
*Joto la watu*	Contagion with carnal heat
*Kifafa*	Seizures as in epilepsy, grand mal
*Kubanua mbavu*	Ribs spreading apart, chest in-drawing
*Kulegea*	Weakened, soft, as overripe fruits
*Kupauka*	Paleness, to become pale
*Kustuka*	To jerk, startle
*Mashetani*	Evil spirits, scare infants from breastfeeding
*Mchango*	Literally 'worms', convulsions when used with *Kustuka*
*Pumo*	Difficult breathing, asthma
*Zongo*	Evil eyes, evil eye illness, with breastfeeding failure/vomiting, by bewitchment

*Homa *signifies febrile illness of any kind, *joto kali *high fever, and *homa kali *includes strong febrile illness sometimes with vomiting, diarrhoea and/or convulsions. *Mchango *is illness with convulsions, accompanied or starting with *kustuka*, meaning startled, frightened or shocked. *Mchango *is feared, because if untreated it may develop into *kifafa*, grand mal seizures of epilepsy, but this can be avoided by herbal prevention and treatment given during symptoms and at each following new moon, as tea, baths and fumigations of mother and child. *Kulegea*, weakened body, means becoming soft, as an overripe fruit, and is often accompanied by vomiting and diarrhoea. When breast-milk is vomited or not taken the milk is considered bewitched by a person with evil eyes: a form of witchcraft called *zongo*, which is also the name of the illness. The healer treats this by cleansing the mother together with her baby, who is given purgatives. *Zongo *is much feared, and speaking about it was a matter of trust,, and it took time for the investigator to realize and adapt to this. However, the regular visits and long-term investigation over more than a year made it clear that *zongo *was frequent, and that *zongo *is an important danger sign recognizing prostration or lethargy, cf. Table 2. *Kulegea *was also sometimes thought to be caused by too strong (*kali*) anti-malarials, as a side-effect of quinine, amodiaquine or sulfadoxine-pyrimethamine.

**Table 2:** Mothers'health seeking behaviour during presentation of danger signs in infants.

Mothers' report in *Kiswahili *on danger signs	English translation and details on signs, in no. of infants	No. of infants admitted to hospital exclusively	Infants exclusively*** treated traditionally	Infants both admitted and treated traditionally	Other****
*Kulegea *only	Weak body, 29*	2	10	6	11
Manifestations of *kulegea*, called *zongo *when severe and treated traditionally	Breastfeeding failure, too weak to sit/be carried, sleep all time, prostration/lethargy, 25*	5	14	4	2
*Mchango*	Convulsions,19 *	1	12	2	4
*Kupauka*	Paleness**, 21*	5	7	5	6
N.a.	None of above signs, 21	3	3	0	15
Total	63	11	24	9	19

*In most instances, more than one symptom or sign occurred simultaneously

**Paleness is counted as a danger sign, but was in fact only responded to in action by mothers, when other signs were also occurring

*** Exclusively does not exclude simultaneous or sequential combination of traditional treatment with out-patient treatment or allopathic home treatment as a past or second choice. However, these were at stages during the illness progression when mothers saw this as *homa *or uncomplicated malaria, and not during danger signs.

**** Other includes out-patient treatment, home-treatment with allopathic medicine, failure to get treatment, or illness regarded as self-limiting.

### Anaemia development and mothers' recognition of 'lack of blood'

Among the 63 infants Hb tested, 24 developed moderate-to-severe anaemia, as defined as Hb < 8 g/dl, and six Hb < 7 g/dl, though none below 5 g/dl. Because Hb was only estimated as part of the study on three occasions, correlation between actual level of Hb and development of symptoms and signs potentially related to anaemia (low Hb) had to be reflected in pro- as well as retrospect; occasionally Hb was known from unplanned visits to health facilities. The median time between symptom appearance, reported by mothers, and actual screening for Hb was five weeks. A total of 21 infants developed moderate-severe anaemia (Hb < 8 g/dl) within six weeks from mothers' recognition of illness. Mothers' knowledge on 'malaria', fever and 'anaemia' and possibly related symptoms and signs were only asked for during an actual illness episode, and general interviews on malaria were deliberately not undertaken, to avoid induction of answers and knowledge bias, as indicated above. Interestingly, the *Kiswahili *word for anaemia *upungufu wa damu*, meaning lack of blood, was mainly used by hospitals as a diagnosis, while mothers were hesitant to suggest themselves that their child lacked blood, even if most had visited health facilities. However, some learning experiences of attending health facilities were reflected in mothers, who knew how to look for paleness of the body, *kupauka*, and considered *kupauka *as a condition arising from *homa kali*, strong fever (Table 1).

### Danger sign recognition, health seeking behaviour, and illness interpretation

About two thirds of the 63 children had at one point suffered from what mothers considered severe manifestations: *kulegea *(weakened or soft body)*, mchango *(convulsions), *zongo *(evil eyes with breastfeeding failure, 'spoilt milk') and combinations hereof or other symptoms, including pallor. The main manifestations and actions taken by mothers appear from Table 2.

Mothers' recognition of symptoms lead to urgent action in a vast majority of cases, but 52 % went for traditional treatment plus minusallopathic ('western') health care. For manifestation mothers considered severe, almost three of every four mothers used traditional healing. Among 30 taking herbal/traditional treatment, 70% did so in conjunction with allopathic treatment with anti-malarials, at clinic or hospital.

Paleness was recognized on 21 occasions by mothers, in 14 instances within six weeks of actual Hb estimation, which proved that 10 children in fact had low Hb; this manifestation was often accompanied by other manifestations, as indicated in Tables 2 &3. But by itself pallor did not lead to action; this was only taken if other signs were also seen.

**Table 3:** Mothers' reports of danger signs in infants, and anaemia development, as confirmed by Hb testing (within 6 weeks).

Mothers' report on danger signs, in English translation, during14 months	Total no. of infants with symptom/sign and Hb testing +/- 6 weeks, ***a***	No. of infants with moderate-severe anaemia, i.e. Hb< 7 g/dl, (% of ***a***)
Weak body, 29*	19*	11* (58)
Breastfeeding failure, too weak to sit/be carried, sleep all time, prostration/lethargy 25*	17*	14* (82)
Convulsions, 19*	8*	6* (75)
Paleness, 21*	14*	10* (71)
None, other 21*	39*	6* (15)
Total 63	63	21 (33)

*In most instances, more than one symptom or sign occurred simultaneously

Furthermore, there was an increasing correlation between low Hb and general danger signs/symptoms, from being soft/weak and unable to sit on back, over breastfeeding failure to prostration and convulsions: from 58 to 82 % of infants had low Hb with presence of these manifestations, recognized early by their mothers.

### An algorithm for home-based anaemia recognition using general danger signs

Maternal reports of general danger signs, besides of paleness, were significantly associated with present or developing moderate-severe anaemia. Those signs indicated in Table 3 with the highest percentage of presence when low Hb, i.e. breast-feeding failure, prostration/lethargy and/or convulsions, in Kiswahili *kulegea, zongo, and mchango*, were pooled and are given in Table 4 in relation to presence and development of anaemia.

**Table 4:** Sensitivity, specificity and significance of maternal report of general danger signs in infants, to detect present anaemia§, and developing anaemia§§

Indicator	Present anaemia	Anaemia development
Sensitivity	15/21 (71 %)	20/24 (83 %)
Specificity	36/42 (86 %)	25/39 (62 %)
Significance of correlation	P < 0.00001	P = 0.0005

*Prostration/lethargy, breastfeeding failure or convulsions

§Hb < 8 g/dl +/- 6 weeks from home-visit; §§Hb < 8 g/dl; anaemia within total follow up period.

Thus, in more than 2/3 of infants with breast-feeding failure, prostration/lethargy and/or convulsion, anaemia was imminent, and should be addressed when managing infants and young children, whether febrile or not. So far, the problem is indeed addressed by the mothers, but not as anaemia.

## Discussion

The present study confirms that symptoms and signs that may be related to malaria, including to anaemia are frequent among infants in Muheza town. Muheza district is an area of intense malaria transmission [19], where the infant mortality rate is 133/1,000 or > 25 % higher than the national average for Tanzania [20]. In spite of having been a field site for numerous malaria research studies for decades [21], and the availability of a designated hospital with a MCH-clinic and three dispensaries, one public and two private, as well as three private pharmacies, it appears that health seeking behaviour is often towards traditional healers and herbal medicine. A previous study in Muheza district [20] revealed that only 1/3 of febrile infants and young children in fact got in contact with the allopathic health system. Accordingly, herbal medicines are sold at the town market, and in the district, and each neighbourhood in town has a number of traditional healers.

It is encouraging that in spite of more frequent use of traditional versus allopathic health systems, mothers were able to identify danger symptoms and signs early, and took action against them within 24 hours, as described in details elsewhere (Ringsted, in preparation). As expected, paleness was less useful to indicate present or emerging low Hb, while more general danger signs, with well-known significance in the local language, *Kiswahili*, particularly prostration/lethargy, unable to sit or to be carried on mothers back, or sleeping all time. These signs, designated *kulegea *or *zongo*, or convulsing, called *mchango*, appeared useful entry points for mothers' recognition, and action, and – in future – for health education and potential inclusion in the local Integrated Management of Childhood Illness (IMCI) algorithms, together with the less sensitive and specific pallor or paleness; *kupauka*. It should be noted that most mothers did not know or use the vocabulary used by local health staff for anaemia, namely *upungufu wa damu*.

The present study was not undertaken to try correcting what may be considered mothers' misconceptions and superstition regarding causes of infant illnesses, but it underscored that it is not only cerebral malaria-related symptoms such as *degdege *that are taken primarily to traditional healers, as others have indicated [11-14]. When attempting to integrating management of illnesses in young children, it should be born in mind that mothers are the first to recognize danger signs. Taking their observations seriously and integrating them into realistic, and not only rational programmes is consequently a precondition for improved care and reduced mortality and morbidity. Others have shown in neighbouring Kenya [7] that parents may recognize very severe anaemia (< 5 g/dl) only, and that combining with other signs, including poor feeding resulted in lower accuracy. That study, however included older children, from thousands of households, but not over 14 months, longitudinally, and prospectively. Accordingly, these authors recommended prospective community-based studies, such as this one. Whether these findings may be of use outside low-land Tanzania remains to be shown.

## Conclusion

This longitudinal, prospective ethnographic study found that mothers to infants in Muheza town, a high-transmission area, with anaemia being the main manifestation of malaria, and where the infant mortality remains high in spite of decades of research and interventions, are able to recognize danger signs related to malaria, including anaemia, early on, and to take treatment seeking action, often within 24 hours. By using the local joint language, *Kiswahili*, it was found that the following signs: weakness, and more specific and sensitively, prostration/lethargy, inability to sit or be carried on mothers' back, or convulsions, correlated well with actual or developing low Hb. Furthermore, it was found that in cases where mothers feared that their children suffered from the much feared *zongo *or *mchango*, they mainly consulted traditional healers, who treated both mother and child. Mothers' concepts and observations deserve being incorporated in the local algorithms for integrated management of infant and young child illness, and traditional healers whom are often approached before or along with the established allopathic health system should be considered to be integrated as well, to help reducing the persistent high infant mortality in the area.

## Authors' contributions

FR planned and carried out the study and wrote 1.st draft of manuscript

ICB outlined the 2.nd version of the manuscript, and together with

HS helped designing the study and analysing the data, and supervised it.
